# Tele-Healthcare in Research and Education: Age Differences in Access and Utilization of Healthcare Resources

**DOI:** 10.1093/geroni/igab046.3642

**Published:** 2021-12-17

**Authors:** William Hills, Matthew Murphy, Karen Hills

**Affiliations:** 1 Coastal Carolina University, Conway, South Carolina, United States; 2 Beaufort Jasper Hampton Comprehensive Health Services, Conway, South Carolina, United States

## Abstract

Societal needs highlighted during the pandemic have led to significant changes in healthcare, including the rapid development and implementation of tele-care consumer options. This study examined video-based, virtual healthcare access and utilization before and during the pandemic. Participants included traditional college-aged students, middle-aged adults, and retirement-aged persons (n = 685); measures included access to physical and mental health services, consumer satisfaction with type of services accessed, and anticipated use of virtual healthcare following the pandemic. Results showed that approximately half of participants (49.2%) had experience with virtual healthcare, with most of these cases during the pandemic. Virtual healthcare was more often used for physical compared to mental healthcare services, with college-aged participants more likely to use mental healthcare services than adult and retirement-aged participants. Laptop computers were most widely used to access services, with smartphone use proportionally lower in retirement-aged participants (31.4%). Overall satisfaction with virtual services was high (Mdn = 5 on a 6-point Likert scale), but college-aged participants trended toward a lower satisfaction (Mdn = 4.25) than other age categories. These results support that virtual healthcare service development and access experienced significant growth during the pandemic. Age differences in the types of services, types of devices, and satisfaction with virtual services all suggest a similar theme for age-related considerations of life stage, life responsibilities, and comfort and familiarity with technology that must be addressed for virtual healthcare to reach its full potential and reach equitably across the lifespan.

